# High level of miR-196b at newly diagnosed pediatric acute myeloid leukemia predicts a poor outcome

**DOI:** 10.17179/excli2016-707

**Published:** 2017-03-07

**Authors:** Lihua Xu, Yang Guo, Wenying Yan, Jiannong Cen, Yuna Niu, Qing Yan, Hailong He, Chien-Shing Chen, Shaoyan Hu

**Affiliations:** 1 Department of Hematology and Oncology, Children's Hospital of Soochow University, Suzhou 215003, Jiangsu, China; 2 Department of Pediatrics, Shanghai East Hospital, Tongji University School of Medicine, Shanghai 200120, China; 3 Center for Systems Biology, Soochow University, Suzhou, 215006, Jiangsu, China; 4 The First Affiliated Hospital of Soochow University, Suzhou, 215006, Jiangsu, China; 5 School of Laboratory Medicine, Xinxiang Medical University, Xinxiang, 453003, Henan, China; 6 Department of Internal Medicine, Division of Hematology and Medical Oncology & Biospecimen Laboratory, Loma Linda University, Loma Linda, CA 92350, USA

**Keywords:** miR-196b, AML, pediatric/child, biomarker

## Abstract

Differential expression of microRNAs (miRNAs) has been implicated in leukemogenesis. We investigate the expression pattern of miR-196b. Using quantitative real-time PCR (qRT-PCR), we detected the expression of miR-196b and its correlated genes (SMC1A/MLH1) in initial pediatric AML. A significant association was observed between overexpression of miR-196b and inferior overall survival of pediatric AML (Log Rank *P*<0.0001). AML M4/5 subtype, high white blood cell (WBC) count at presentation, MLL rearrangement, or FLT3-ITD mutation at diagnosis and non-remission group after the first induction chemotherapy possessed higher miR-196b expression. Furthermore, a positive relationship was found between the expression of miR-196b and SMC1A/MLH1 (Spearman's *r*=0.37 and 0.44, *P*=0.001 and <0.0001, respectively). Taken together, these findings suggest that differentially high expression of miR-196b in diagnostic marrow samples of pediatric AML is associated with unfavorable outcome, and miR-196b potentially can be a novel biomarker for the diagnosis, prognosis and treatment in pediatric AML.

## Abbreviations

qRT-PCR = quantitative real-time reverse transcriptase-polymerase chain reaction, AML = acute myeloid leukemia, POMA = Pipeline of Outlier microRNA Analysis, NOD = number of degree, TFP = transcription factor percentage, ALL = acute lymphoblastic leukemia, BM = bone marrow, MNCs = mononuclear cells, WBC = white blood cell, OS = overall survival

## Introduction

Acute myeloid leukemia (AML) is a highly heterogeneous hematopoietic malignancy with increasing identified cytogenetic and molecular abnormalities (Valk et al., 2004[31]). Comparing to adult AML, pediatric AML has certain unique biologic properties and genetic abnormalities, such as specific karyotype (e.g. Trisomy 21), controversial role of C-kit mutation, and fewer secondary AML, with favorable response to chemotherapy and significantly different prognosis (Creutzig et al., 2012[6]). Over the past few decades, the five-year survival rate of childhood AML improved from around 40 % to 60 %-75 % (Armendariz et al., 2005[1]; Pui et al., 2011[25]; Smith et al., 2005[29]; Kaspers and Creutzig, 2005[11]), benefiting from risk-stratified therapy (Pui et al., 2011[25]; Li and Xiao, 2008[13]). Approximately 90 % of AML children achieve remission with the combination chemotherapy, but relapse (about one third) remains the most significant risk in pediatric AML (Pui et al., 2011[25]; Creutzig et al., 2012[6]). Hence, deeper understanding molecular mechanism of AML is desperately needed, which can potentially further identify targets for effective novel therapies (Moore et al., 2013[20]).

MicroRNAs (miRNAs) are small non-coding RNAs consisting of ~22 nucleotides, with highly conserved sequence in vertebrates (Lagos-Quintana et al., 2001[12]), which are involved with many cellular processes such as proliferation, differentiation, and apoptosis at the post-transcription level (Lujambio and Lowe, 2012[15]). Also, as described having oncogenic or anti-oncogenic properties, miRNAs are implicated in leukemogenesis and the prognosis, due to some microRNA genes locating in regions of translocations and deletions frequently occurring in leukemia (e.g. miR-15a-miR-16-1 cluster and chronic lymphocytic leukemia) (Chen, 2005[4]). Moreover, miRNA expression profiles could classify specific cancers such as discriminating acute lymphoblastic leukemia (ALL) from AML (Mi et al., 2007[19]).

In this pilot study, we compared the microarray expression data of miRNAs and mRNAs from pediatric AML, reported from the National Center for Biotechnology Information comprehensive gene expression database (NCBI GEO database, accession number: GSE35320 and GSE43176). Based on the previous published novel prediction software POMA (Pipeline of Outlier microRNA Analysis) (Zhang et al., 2014[35]), we made further improvement to get a specific microRNA-mRNA network for pediatric AML in order to explore relevant biomarkers (Yan et al., 2015[34]). Through network analysis, miR-196b was identified as one of the candidate miRNA biomarkers involved in the leukemogenesis of childhood AML, due to a significantly larger NOD (number of degree) and TFP (transcription factor percentage) (Wilcoxon test, p<0.05, NOD=30, TFP=0.191) (Zhang et al., 2014[35]; Yan et al., 2015[34]). Overexpression of miR-196b has been reported in 71 European pediatric AML with MLL gene rearrangements, NPM1 mutations, as well as FLT3-ITD in cytogenetically normal background (Danen-van Oorschot et al., 2012[7]). To further explore and confirm the potential function of miR-196b, we investigated the expression of miR-196b and its possible relevant genes SMC1A/MLH1 in 83 Chinese pediatric AML. Quantitative real-time reverse transcriptase-polymerase chain reaction (qRT-PCR) was performed on bone marrow samples and cell lines.

## Methods

### Cell culture

Human myeloid leukemia cell lines, HL60, NB4, MV4-11, SHI1, Kasumi-1, and K562, were maintained at 37 °C and cultured in RPMI-1640 (Hyclone) supplemented with 10 % fetal bovine serum (FBS) (Bovogen) in 5 % CO_2_ humidified atmosphere. SHI-1 cell line was cultured in IMDM (Hyclone) supplemented with 20 % FBS.

### Patient samples

112 bone marrow (BM) specimens from 83 initial pediatric AML and 29 non-malignancies with age ≤ 18 years were included. All specimens were collected randomly before treatment from January 2012 to October 2014 in the Affiliated Children's Hospital of Soochow University with informed consent. Mononuclear cells (MNCs) were isolated and stored at -80 °C prior to RNA extraction. AML children were treated according to the recommendations for Chinese AML children (Subspecialty Group of Hematology Diseases et al., 2006[30]). Of 83 AML patients, 63 patients were classified as “chemotherapy” group and were included in survival analysis, while 20 patients eventually selected hematopoietic stem cell transplantation. Follow-up time started from chemotherapy to death or default of patients. Twenty-nine BM specimens with benign conditions were used as controls, including infectious disease (6/29), healthy donor (3/29), and idiopathic thrombocytopenic purpura (20/29). The study was approved by the ethics committee of the Affiliated Children's Hospital of Soochow University. 

### Measurement of miR-196b and SMC1A/ MLH1 gene expression in clinical samples and cell lines

TaqMan probe based qRT-PCR method was applied to detect the expression of miR-196b and U6 in each bone marrow specimen and six cell lines. MiR-196b level was normalized using U6 as a housekeeping gene. All small RNA primers and the TaqMan probes were designed and supplied by Applied Biosystems (ABI) (miR-196b: No. 002215-PN4427975; U6: No. 001973-PN4427975). SMC1A and MLH1 gene were measured on mRNA level by SYBGreen based qRT-PCR with β-actin as internal control. Primers of three mRNAs were designed by Primer Premier Software (version 5.0) and synthesized by Sangon Biotech (Shanghai). The counterpart sequences are given in Table 1(Tab. 1).

### Total RNA isolation and cDNA synthesis

Total RNA was extracted using the Trizol reagent (Invitrogen, China). RNA quality was checked on MULTISKAN GO (Thermo Scientific, China). Reverse transcription (RT) reactions were carried out by PCR System 9700 GeneAmp under each specification. Reaction system (15 µl) and parameters of miR-196b and U6 were set according to the protocol of ABI, while those of SMC1A/MLH1/β-actin were set as: 40 μl of sample volume; 70 °C for 5 min (preheat), then 37 °C for 60 min, 95 °C for 5 min, and 4 °C forever, for one cycle. 

### Real-Time Quantitative RT-PCR

qRT-PCR was executed on ABI 7500 Real-Time PCR System. MiR-196b and U6 were detected according to the manufacturer's procedure from ABI. SYBGreen based qRT-PCR for SMC1A, MLH1, and reference gene β-actin were executed based on the following reaction volume: cDNA 4 μl, SYBGreen 1 μl, Mix 12.5 μl, Primer F 0.5 μl, Primer R 0.5 μl, and Nuclease-free water 6.5 μl. Reaction parameters were set as: 50 °C 2 min, 95 °C 10 min, 95 °C 20 sec, 56 °C 20 sec, 72 °C 45 sec, 95 °C 15 sec, 60 °C 60 sec, 95 °C 15 sec, 60 °C 15 sec, 25 μl of reaction system, and 40 cycles. Melting curves were drawn automatically by computer.

Triplicates were performed for all qRT-PCR reactions. Ct threshold was manually set at 0.08. Ct value of housekeeping gene U6 and β-actin should meet the requirement of less than or equal to 25 and 18, respectively. The quantitative PCR values of all samples were normalized to those of housekeeping gene. Using the comparative Ct method, the gene relative expressions of 112 samples were calculated to median expression in controls and expressed in 2^-ΔΔCt ^(Livak and Schmittgen, 2001[14]). 

### Statistical analysis

All the statistical analyses and plotting were performed by SPSS 18.0 and Graphpad Prism 5.0 software package. Statistical differences of genes expression between groups were calculated using the Mann-Whitney and Kruskal-Wallis test. Spearman's nonparametric correlation analysis was used for comparison between genes expression and clinical features. Kaplan-Meier survival analysis was used to assess the impact of gene level on the survival time (overall survival, OS). All used test were two-sided, *P*-valueless than 0.05 was considered as statistically significant.

## Results

Patients’ clinical characteristics, cytogenetic and molecular abnormalities were listed in Table 2(Tab. 2). Based on World Health Organization (WHO) classification 2008 (Vardiman et al., 2009[32]) and NCCN Guidelines for AML (NCCN, 2015[22]), patients were stratified into diverse subgroups (Table 3(Tab. 3)).

### Differential expression of miR-196b is significantly associated with specific subgroups (FAB classification)

**Figure 1:**
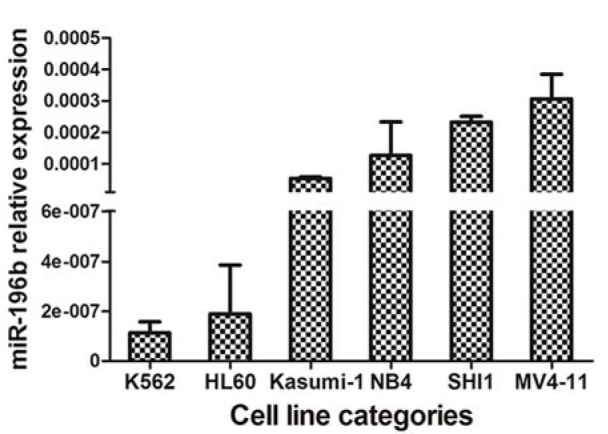
Differential expression of miR-196b in myeloid leukemia cell lines (HL60, NB4, MV4-11, SHI-1, Kasumi-1, and K562). Vertical lines indicate the expression of miR-196b relative to β-actin in cell lines. Obviously higher levels were found in MV4-11 and SHI-1.

**Figure 2:**
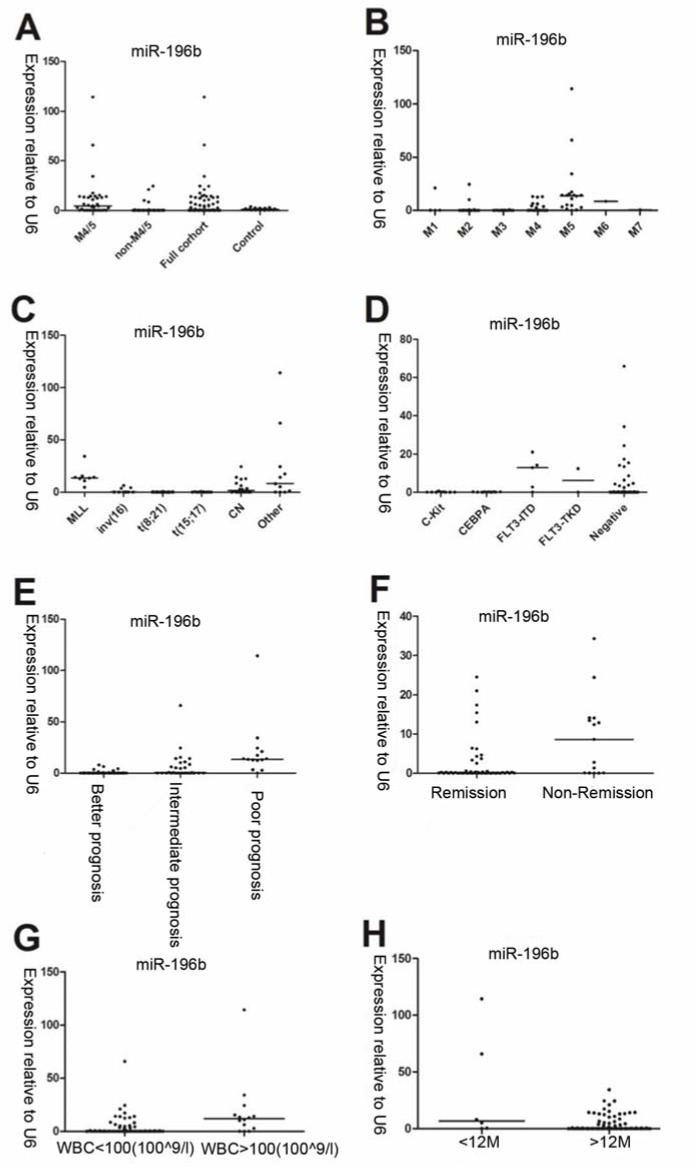
The relative expression of miR-196b determined by qRT-PCR is related to specific FAB (French-America-British) classification, cytogenetic/molecular aberrations, and general clinical characteristics in pediatric AML. A: Expression of miR-196b is higher in M4/5 and lower in non-M4/5 subgroup as compared to controls. B: Differential miR-196b expression exists in diverse FAB subtypes. C: Differential expression presents in various cytogenetic subgroups in 79 AML children. D: Expression in relation to different molecular aberrations detected in 61available patients. E: Overexpression of miR-196b is associated with unfavorable outcome. F: Expression of miR-196b is higher in remission group than non-remission group. G: Expression of miR-196b is higher in WBC ≥ 100×10^9^/L than in WBC < 100×10^9^/L cohort. H: Higher expression of miR-196b appeared in age ≤ 12M than age > 12M

We first tested leukemia cell lines and demonstrated significantly higher expression of miR-196b in MV4-11/SHI-1 but relatively lower in HL60 cell lines, suggesting miR-196b was not consistently expressed among different leukemia lines (Figure 1(Fig. 1)). In primary leukemia clinical samples, the level of miR-196b was significantly higher in M4/5 than that in non-M4/5 subgroup with over 62-fold difference (*P*<0.0001). Both M4/5 (monocytic AML series) and non-M4/5 (non-monocytic AML series) subgroup had statistically differential expression compared to those in controls (*P*=0.032, <0.0001, respectively) (Table 3(Tab. 3), Figure 2A-B(Fig. 2)). 

### Overexpression of miR-196b correlated with prognostic factors and low remission rate after the first induction chemotherapy 

Among abnormal cytogenetic subgroups, expression level of miR-196b was the lowest in t (8; 21) (n=22) and the highest in MLL-rearrangement (n=8) compared with other cytogenetic subgroups (*P*=0.0002, *P*=0.0002, respectively). Furthermore, miR-196b expressions were significantly different among various molecular mutation subgroups, with the highest expression in FLT3-ITD (*P*=0.04), and the lowest level in C-Kit mutation subgroup (*P*=0.06) (Table 3(Tab. 3), Figure 2C-D(Fig. 2)).

The highest level of miR-196b was found in poor prognosis group while the lowest expression in favorable prognosis group (*P*<0.0001, *P*<0.0001, respectively). MiR-196b expression was significantly higher in non-remission group (n=15) as compared to remission group (n=51) after the first induction remission therapy (*P*=0.020). In addition, the expression of miR-196b in WBC ≥ 100×10^9^/L cohort was statistically higher compared with WBC<100×10^9^/L cohort (*P*=0.004), and no obvious relationship was observed between miR-196b expression and blast percentage of PB/BM (*P*>0.05). Furthermore, statistical difference in miR-196b also existed between age ≤ 12 months and > 12 months subgroups (*P*=0.029). No significantly difference was observed between genders (*P*=0.141) (Table 3(Tab. 3), Figure 2E-H(Fig. 2)).

In summary, miR-196b expression is highly correlated with known poor prognostic factors in AML, and patients with overexpressed miR-196b are not easy to achieve remission.

### Correlation analysis of miR-196b levels with clinical characteristics and outcome of pediatric AML

**Figure 3:**
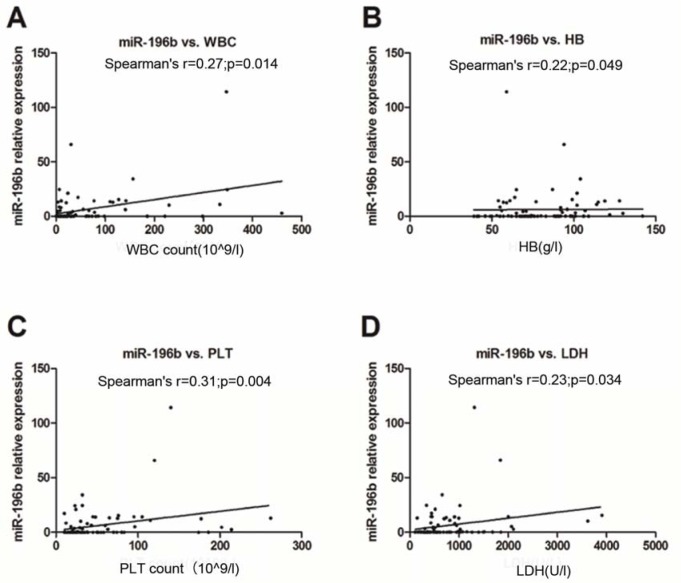
Correlation analysis of miR-196b expression with primary WBC (A), HB (B), and PLT (C) count of peripheral blood (PB) as well as serum LDH value (D)

Using Spearman's correlation analysis, we compared the relationship of miR-196b levels with presenting clinical features, and found that miR-196b expression was positively associated with high WBC, hemoglobin (HB), and platelet (PLT) count as well as serum lactate dehydrogenase (LDH) value (Spearman's *r*=0.27, 0.22, 0.31, 0.23, *P*=0.01, 0.04, 0.004, 0.034, respectively) (Figure 3A-D(Fig. 3)). No significant correlation between the expression and serum C-reaction protein (CRP) was noted (Spearman's *r*=0.22, *P*=0.052).

**Figure 4:**
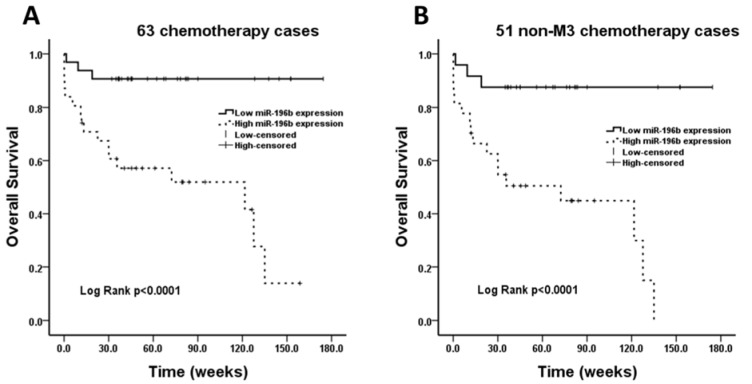
Survival analysis performed in chemotherapy group. A: The overall survival rate is statistically higher in low miR-196b expression group compared with high miR-196b expression group in 63 cases. B: After removing 12 patients with M3 subtype, the overall survival rate in low miR-196b expression group is still obviously higher than that in high miR-196b expression group.

To evaluate the association of miR-196b level with outcome of AML patient, survival analysis was performed for “chemotherapy” group (n=63). The median follow-up was 60.4 weeks, and follow-up rate was 97 %. 63 cases were divided into low miR-196b expression (≤ median) and high miR-196b expression (> median) subgroups. Significant correlation was found between low miR-196b expression and improved outcome (OS) (Log Rank *P*<0.0001) (Figure 4A(Fig. 4)). After removing twelve cases with M3 subtype, statistical correlation was still observed (Log Rank *P*<0.0001) (Figure 4B(Fig. 4)). 

### Overtly differential overexpression in SMC1A and MLH1 compared to controls, and positive correlation with the expression of miR-196b

**Figure 5:**
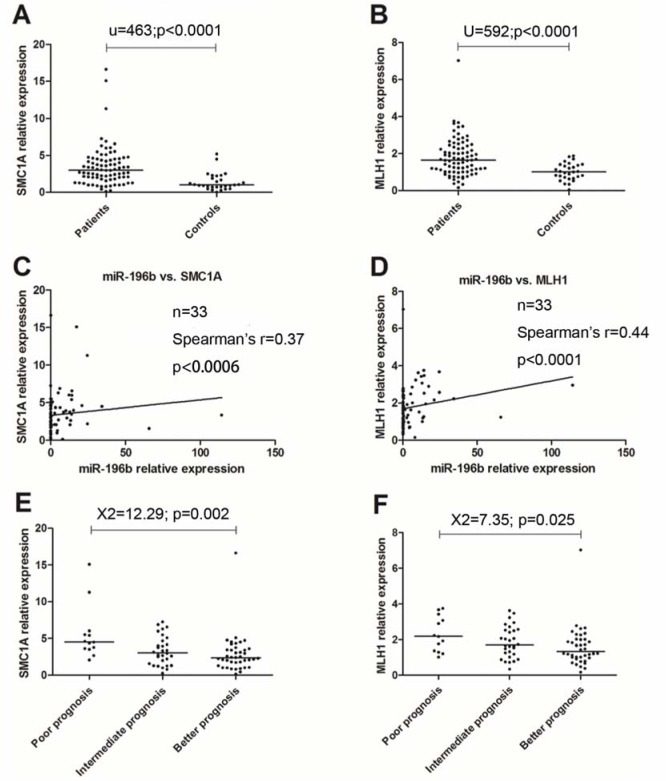
qRT-PCR results for SMC1A/MLH1. The expression of SMC1A/MLH1 is high expression as compared to controls, and positively correlates to that of miR-196b. Adverse relevance similar to miR-196b was also found in prognosis groups, and cytogenetic groups.

SMC1A and MLH1 were studied in 112 samples. Among them, eighty three were newly diagnosed AML. The levels of SMC1A/MLH1 were found significantly higher as compared to controls (*P*<0.0001, <0.0001, respectively) and positively correlate with miR-196b (Spearman's *r*=0.37, 0.44; *P*=0.001, <0.0001, respectively) (Figure 5A-D(Fig. 5), Table 4(Tab. 4)).

We further compared the expression levels of SMC1A/MLH1 among diverse clinical subgroups. Statistical difference was also found among three prognosis groups (*P*=0.002, 0.025, respectively) (Figure 5E-F(Fig. 5)).

However, no obvious correlation was observed between SMC1A/MLH1 expression and OS of patients in neither 63 chemotherapy group (Log Rank P>0.05) nor 51 non-M3 chemotherapy group (Log Rank P>0.05).

## Discussion

In this exploratory study on the expression of miR-196b in newly diagnosed pediatric AML in China, we found that miR-196b expression was significantly higher in M4/5 (FAB classification) AML, and strongly linked to initial high WBC, early treatment response, unfavorable chromosome karyotype (11q23), and molecular abnormalities (FLT3-ITD mutation), as well as inferior clinical outcome (OS). MiR-196b might be a useful prognostic predictor in Chinese pediatric AML.

MiR-196b, which belongs to miR-196 gene family (miR-196a-1, miR-196a-2, and miR-196b), has been reported to inhibit cell apoptosis and promote cell proliferation in a myeloid leukemia cell line THP-1 (Cao et al., 2015[3]). In adult AML, higher levels of miR-196b not only found in MLL-rearranged AML (Popovic et al., 2009[24]), but in total patients compared to healthy donor (Coskun et al., 2011[5]). Moreover, the adverse association of high miR-196b expression with OS has been demonstrated on 53 AML (median age: 49 years) and 238 adult AML reported in literature (Wang et al., 2010[33]; Diaz-Beya et al., 2014[8]). Consistent with these, in the present study aimed to pediatric AML, we also found similar results for OS related miR-196b level. In addition, we showed higher expression of miR-196b in FLT3-ITD mutation, or M4/5 subtype, which are widely recognized to have poor outcomes in clinic. Besides, we also found a relatively lower expression of miR-196b in patients with CEBPA mutation, t (8; 21) subgroup, which have favorable prognosis (Creutzig et al., 2012[6]). Taken together, these data directly or indirectly suggested the potentially prognostic value of miR-196b. Initially differential expression of miR-196b is expected to be a novel biomarker for diagnosis of specific subtype and prognosis in pediatric AML.

Nevertheless, certain discrepancies were also presented compared with published data. Here, the expression of miR-196b on the total level was slightly reduced instead of increasing, which is other than previous studies on adult AML (Coskun et al., 2011[5]). One of reasons might be that the granulocyte series (M2) patients made up the main part of the entire cohort (Table 2(Tab. 2)). We also found a lowest level of miR-196b in patient with C-Kit mutation compared with others. Earlier studies have found that C-Kit mutation is associated with unfavorable prognosis of adult AML with t (8; 21) ( Boissel et al., 2006[2]), and AML children with t (8;21) or CBF-rearrangement (Shimada et al., 2006[28]; Manara et al., 2014[17]), yet other investigations indicated that no significant relevance existed between C-Kit mutation and the prognosis of AML children and adults (Pollard et al., 2010[23]; Goemans et al., 2005[9]; Shih et al., 2008[27]; Riera et al., 2013[26]). As is mentioned before, we forcefully demonstrated the prognostic relevance of low miR-196b expression to favorable outcome. Thus, the results raised the possibility that, C-Kit mutation may not necessarily be associated with poor outcome in pediatric AML. These disputes await more in-depth answers. In addition, the previous study (Danen-van Oorschot et al., 2012[7]) failed to found obvious correlation between miR-196b expression and OS of pediatric AML as well as to some clinical features. It also differs from our results. We not only showed that the negative correlation of miR-196b to OS but also found the level elevated with the increasing of initial WBC, HB, and PLT count and LDH level of PB. Among them, initial WBC count is the usual prognostic factor in pediatric AML, yet the meanings of HB and PLT are still dismal and under-reported. Its clinical significances remain to be further investigated.

To further investigate the potential role of miR-196b, we studied SMC1A and MLH1 genes, which are key genes involved in two key pathways related to DNA repair and stability (i.e. sister chromosome polymerization and DNA mismatch repair (MMR) pathway respectively), and were reported to contribute to the poor outcome of AML. One study from Homme et al. (2010[10]) demonstrated the correlation of low SMC1A protein with poor prognosis. The other study from Mao et al. (2008[18]) showed that mutated 3'-UTR of MLH1, likely by low MLH1 protein level, could cause AML relapse. In the present study, we failed to meet such results. Instead, we found significantly higher SMC1A/MLH1 mRNA expression in poor prognosis group and entire AML cohort compared to controls. It seems to be different from the previous studies. However, these two researches on solid tumor have demonstrated the overexpression of SMC1A mRNA in cervix cancer and human glioma (Narayan et al., 2007[21]; Ma et al., 2013[16]), which supported our results. Moreover, gene expression levels on mRNA level of SMC1A/MLH1 were different from that on protein level (Homme et al., 2010[10]; Mao et al., 2008[18]). Furthermore, age might be also an important factor, which could confer different expression profile of SMC1A/MLH1 in children. These inconsistencies await deeper interpretation. In addition, we found a robustly positive connection between SMC1A/MLH1 and miR-196b on the mRNA level, under an OS correlation with miR-196b but not SMC1A/MLH1, which might be due to different detection levels (e.g. mRNA or protein) even age-dependent. It also indicated that a complex regulatory network could be involved in miR-196b, SMC1A/MLH1, and the two pathways as mentioned, which needs further investigation.

## Conclusion

In conclusion, we identified the differential expression of miR-196b and its clinical significance in initial pediatric AML. High level of miR-196b is associated with specific FAB subtype, cytogenetic and molecular subgroup, as well as poor outcome. Some limitations can be attributed to individual heterogeneity, relatively small sample size, and shorter observation duration. Prospective study on miR-196b in pediatric AML treatment protocol is warranted.

## Notes

Lihua Xu and Yang Guo contributed equally as first authors. 

## Acknowledgements

The authors would like to thank Hongjie Shen, Naichao Yang, Xuejun Shao for their assistance, and Zixing Chen for helpful discussion and critical reading of the manuscript. This work was supported by the Natural Science Foundation of China (No. 81370627 and NO.81170513), Jiangsu Province key point project (NO. BL2013014), and a Project Funded by the Priority Academic Program Development of Jiangsu Higher Education Institutions.

## Figures and Tables

**Table 1:**
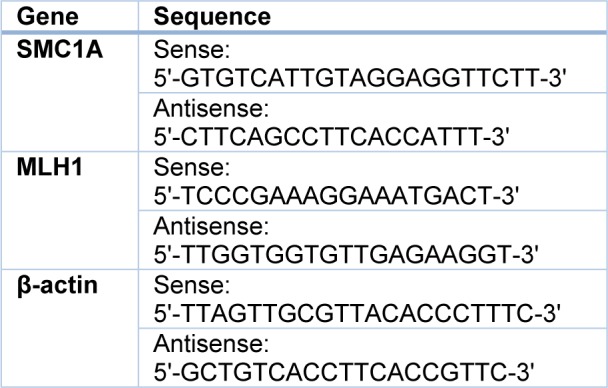
Sequences of genes for SYBGreen based qRT-PCR

**Table 2:**
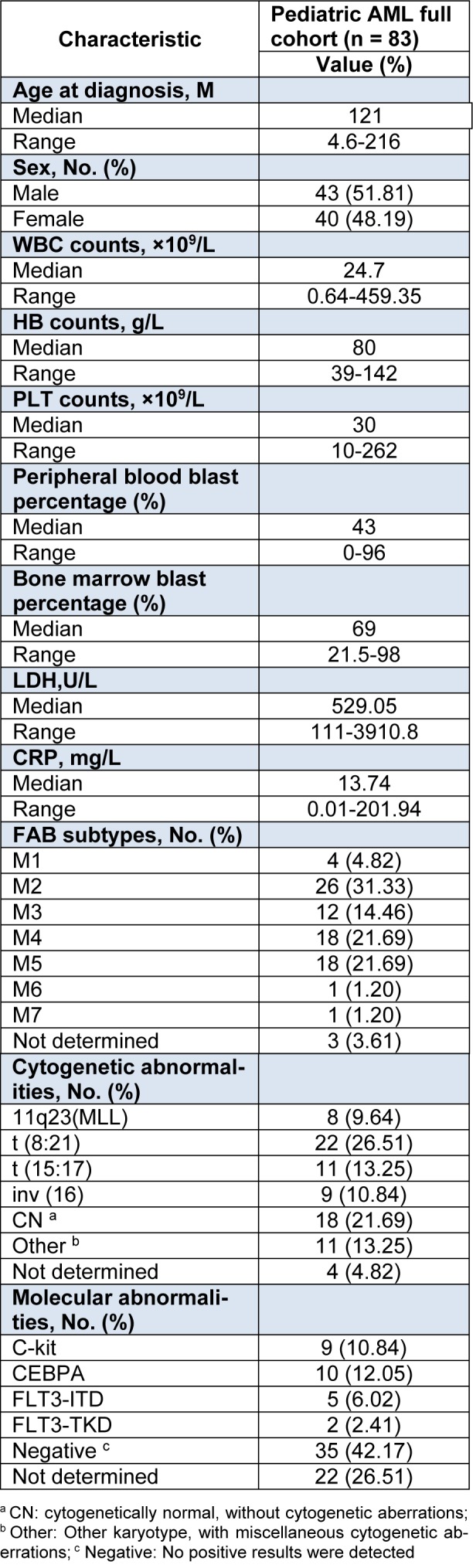
FAB (French-America-British) subtypes and clinical/cytogenetic/molecular characteristics of 83 Chinese pediatric AML patients

**Table 3:**
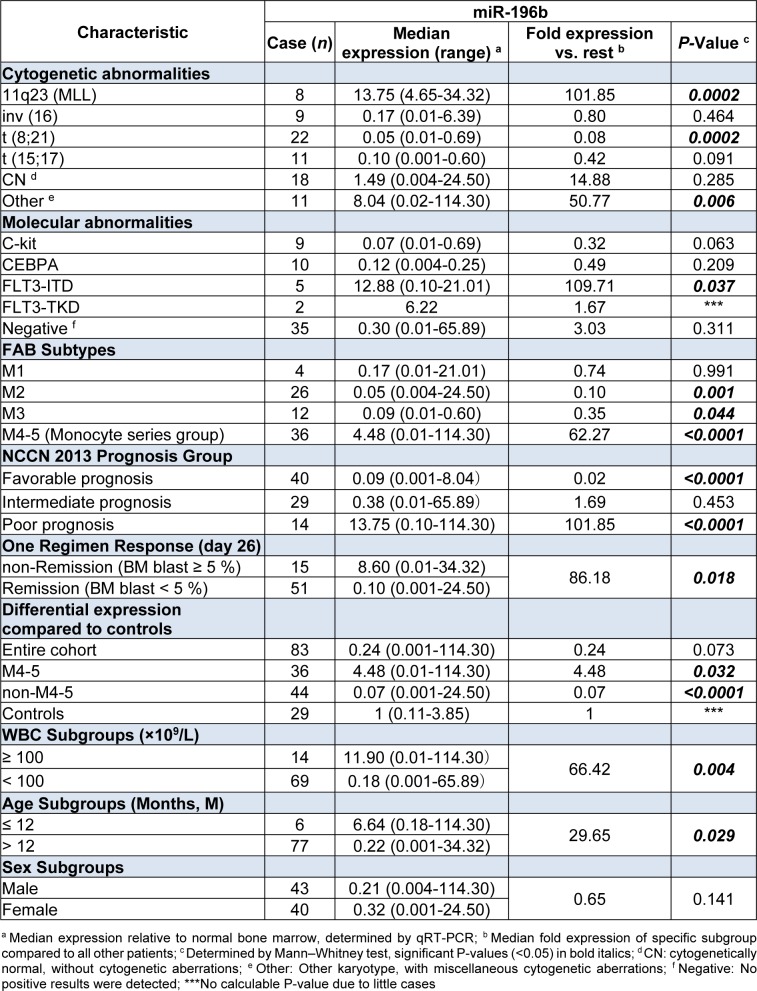
Differential expression of miR-196b among diverse clinical/cytogenetic/molecular subgroups of AML children.

**Table 4:**
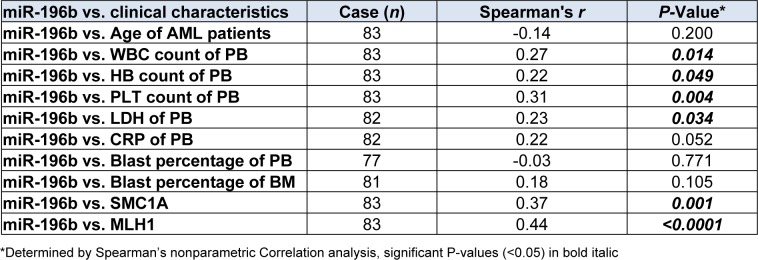
Correlation analysis between relative expression of miR-196b and clinical characteristics of AML children

## References

[1] Armendariz H, Barbieri MA, Freigeiro D, Lastiri F, Felice MS, Dibar E (2005). Treatment strategy and long-term results in pediatric patients treated in two consecutive AML-GATLA trials. Leukemia.

[2] Boissel N, Leroy H, Brethon B, Philippe N, de Botton S, Auvrignon A (2006). Incidence and prognostic impact of c-Kit, FLT3, and Ras gene mutations in core binding factor acute myeloid leukemia (CBF-AML). Leukemia.

[3] Cao D, Hu L, Lei D, Fang X, Zhang Z, Wang T (2015). MicroRNA-196b promotes cell proliferation and suppress cell differentiation in vitro. Biochem Biophys Res Commun.

[4] Chen CZ (2005). MicroRNAs as oncogenes and tumor suppressors. N Engl J Med.

[5] Coskun E, von der Heide EK, Schlee C, Kuhnl A, Gokbuget N, Hoelzer D (2011). The role of microRNA-196a and microRNA-196b as ERG regulators in acute myeloid leukemia and acute T-lymphoblastic leukemia. Leuk Res.

[6] Creutzig U, van den Heuvel-Eibrink MM, Gibson B, Dworzak MN, Adachi S, de Bont E (2012). Diagnosis and management of acute myeloid leukemia in children and adolescents: recommendations from an international expert panel. Blood.

[7] Danen-van Oorschot AA, Kuipers JE, Arentsen-Peters S, Schotte D, de Haas V, Trka J (2012). Differentially expressed miRNAs in cytogenetic and molecular subtypes of pediatric acute myeloid leukemia. Pediatr Blood Cancer.

[8] Diaz-Beya M, Brunet S, Nomdedeu J, Tejero R, Diaz T, Pratcorona M (2014). MicroRNA expression at diagnosis adds relevant prognostic information to molecular categorization in patients with intermediate-risk cytogenetic acute myeloid leukemia. Leukemia.

[9] Goemans BF, Zwaan CM, Miller M, Zimmermann M, Harlow A, Meshinchi S (2005). Mutations in KIT and RAS are frequent events in pediatric core-binding factor acute myeloid leukemia. Leukemia.

[10] Homme C, Krug U, Tidow N, Schulte B, Kuhler G, Serve H (2010). Low SMC1A protein expression predicts poor survival in acute myeloid leukemia. Oncol Rep.

[11] Kaspers GJ, Creutzig U (2005). Pediatric acute myeloid leukemia: international progress and future directions. Leukemia.

[12] Lagos-Quintana M, Rauhut R, Lendeckel W, Tuschl T (2001). Identification of novel genes coding for small expressed RNAs. Science.

[13] Li L, Xiao ZJ (2008). Zhongguo shi yan xue ye xue za zhi.

[14] Livak KJ, Schmittgen TD (2001). Analysis of relative gene expression data using real-time quantitative PCR and the 2(-Delta Delta C(T)) method. Methods.

[15] Lujambio A, Lowe SW (2012). The microcosmos of cancer. Nature.

[16] Ma Z, Lin M, Li K, Fu Y, Liu X, Yang D (2013). Knocking down SMC1A inhibits growth and leads to G2/M arrest in human glioma cells. Int J Clin Exp Pathol.

[17] Manara E, Bisio V, Masetti R, Beqiri V, Rondelli R, Menna G (2014). Core-binding factor acute myeloid leukemia in pediatric patients enrolled in the AIEOP AML 2002/01 trial: screening and prognostic impact of c-KIT mutations. Leukemia.

[18] Mao G, Pan X, Gu L (2008). Evidence that a mutation in the MLH1 3'-untranslated region confers a mutator phenotype and mismatch repair deficiency in patients with relapsed leukemia. J Biol Chem.

[19] Mi S, Lu J, Sun M, Li Z, Zhang H, Neilly MB (2007). MicroRNA expression signatures accurately discriminate acute lymphoblastic leukemia from acute myeloid leukemia. Proc Natl Acad Sci U S A.

[20] Moore AS, Kearns PR, Knapper S, Pearson AD, Zwaan CM (2013). Novel therapies for children with acute myeloid leukaemia. Leukemia.

[21] Narayan G, Bourdon V, Chaganti S, Arias-Pulido H, Nandula SV, Rao PH (2007). Gene dosage alterations revealed by cDNA microarray analysis in cervical cancer: identification of candidate amplified and overexpressed genes. Genes Chromosomes Cancer.

[22] NCCN (2015). NCCN clinical practice Guidelines in Oncology (NCCN Guidelines®). Acute myeloid leukemia version 1. 2015 [EB/OL]. http://www.nccn.org/.

[23] Pollard JA, Alonzo TA, Gerbing RB, Ho PA, Zeng R, Ravindranath Y (2010). Prevalence and prognostic significance of KIT mutations in pediatric patients with core binding factor AML enrolled on serial pediatric cooperative trials for de novo AML. Blood.

[24] Popovic R, Riesbeck LE, Velu CS, Chaubey A, Zhang J, Achille NJ (2009). Regulation of mir-196b by MLL and its overexpression by MLL fusions contributes to immortalization. Blood.

[25] Pui CH, Carroll WL, Meshinchi S, Arceci RJ (2011). Biology, risk stratification, and therapy of pediatric acute leukemias: an update. J Clin Oncol.

[26] Riera L, Marmont F, Toppino D, Frairia C, Sismondi F, Audisio E (2013). Core binding factor acute myeloid leukaemia and c-KIT mutations. Oncol Rep.

[27] Shih LY, Liang DC, Huang CF, Chang YT, Lai CL, Lin TH (2008). Cooperating mutations of receptor tyrosine kinases and Ras genes in childhood core-binding factor acute myeloid leukemia and a comparative analysis on paired diagnosis and relapse samples. Leukemia.

[28] Shimada A, Taki T, Tabuchi K, Tawa A, Horibe K, Tsuchida M (2006). KIT mutations, and not FLT3 internal tandem duplication, are strongly associated with a poor prognosis in pediatric acute myeloid leukemia with t(8;21): a study of the Japanese Childhood AML Cooperative Study Group. Blood.

[29] Smith FO, Alonzo TA, Gerbing RB, Woods WG, Arceci RJ, Children's Cancer Group (2005). Long-term results of children with acute myeloid leukemia: a report of three consecutive Phase III trials by the Children's Cancer Group: CCG 251, CCG 213 and CCG 2891. Leukemia.

[30] Subspecialty Group of Hematology Diseases, Society of Pediatrics, Chinese Medical Association, Editorial Board of Chinese Journal of Pediatrics (2006). Zhonghua Er Ke Za Zhi.

[31] Valk PJ, Verhaak RG, Beijen MA, Erpelinck CA, Barjesteh van Waalwijk van Doorn-Khosrovani S, Boer JM (2004). Prognostically useful gene-expression profiles in acute myeloid leukemia. N Engl J Med.

[32] Vardiman JW, Thiele J, Arber DA, Brunning RD, Borowitz MJ, Porwit A (2009). The 2008 revision of the World Health Organization (WHO) classification of myeloid neoplasms and acute leukemia: rationale and important changes. Blood.

[33] Wang Y, Li Z, He C, Wang D, Yuan X, Chen J (2010). MicroRNAs expression signatures are associated with lineage and survival in acute leukemias. Blood Cells Mol Dis.

[34] Yan W, Xu L, Sun Z, Lin Y, Zhang W, Chen J (2015). MicroRNA biomarker identification for pediatric acute myeloid leukemia based on a novel bioinformatics model. Oncotarget.

[35] Zhang W, Zang J, Jing X, Sun Z, Yan W, Yang D (2014). Identification of candidate miRNA biomarkers from miRNA regulatory network with application to prostate cancer. J Transl Med.

